# Functional Imaging of Audio–Visual Selective Attention in Monkeys and Humans: How do Lapses in Monkey Performance Affect Cross-Species Correspondences?

**DOI:** 10.1093/cercor/bhx092

**Published:** 2017-04-13

**Authors:** Teemu Rinne, Ross S. Muers, Emma Salo, Heather Slater, Christopher I. Petkov

**Affiliations:** 1 Department of Psychology and Logopedics, University of Helsinki, Helsinki, Finland; 2 Advanced Magnetic Imaging Centre, Aalto University School of Science, Espoo, Finland; 3 Institute of Neuroscience, Newcastle University, Newcastle upon Tyne, UK; 4 Centre for Behaviour and Evolution, Newcastle University, Newcastle upon Tyne, UK

**Keywords:** auditory cortex, fMRI, primates

## Abstract

The cross-species correspondences and differences in how attention modulates brain responses in humans and animal models are poorly understood. We trained 2 monkeys to perform an audio–visual selective attention task during functional magnetic resonance imaging (fMRI), rewarding them to attend to stimuli in one modality while ignoring those in the other. Monkey fMRI identified regions strongly modulated by auditory or visual attention. Surprisingly, auditory attention-related modulations were much more restricted in monkeys than humans performing the same tasks during fMRI. Further analyses ruled out trivial explanations, suggesting that labile selective-attention performance was associated with inhomogeneous modulations in wide cortical regions in the monkeys. The findings provide initial insights into how audio–visual selective attention modulates the primate brain, identify sources for “lost” attention effects in monkeys, and carry implications for modeling the neurobiology of human cognition with nonhuman animals.

## Introduction

Although our understanding of the effects of attention on neuronal responses and brain activity is substantial (Hernandez-Peon et al. 1956; Hillyard et al. 1973; Hocherman et al. 1976; Moran and Desimone 1985; Posner et al. 1988; Pugh et al. 1996; Kastner et al. 1998; reviewed in: Fritz et al. 2007; Corbetta et al. 2008; Reynolds and Heeger 2009; Duncan 2010; Alho et al. 2014; Osmanski and Wang 2015), the overriding premise that attention comparably modulates cortical networks in humans and animal models still remains largely untested. Human neuroimaging studies show that attention-engaging tasks are associated with enhanced activations in broad cortical networks consisting of sensory and fronto-parietal regions (Hillyard et al. 1973; Posner et al. 1988; Pugh et al. 1996; Kastner et al. 1998; Yantis and Serences 2003; Fritz et al. 2007; Salmi et al. 2007; Corbetta et al. 2008; Duncan 2010). Neuronal-level studies in animal models, in turn, indicate that attention can increase the fidelity of neuronal responses for attended features (Spitzer et al. 1988; Treue and Maunsell 1999; Briggs et al. 2013), depends on certain neurotransmitter receptors (Herrero et al. 2008; Harris and Thiele 2011) and affects neural synchronization (Fries et al. 2001; Buschman and Miller 2007; Bosman et al. 2012; Lakatos et al. 2016; Michalareas et al. 2016). However, the results of human and animal studies often cannot be directly related to each other, since attention-related modulations are rarely systematically mapped in animals across cortical areas, as they are in humans. Additionally, neural recordings in humans, for instance in patients being monitored for surgery and conducting selective attention tasks are rare (Mesgarani and Chang 2012) and could benefit from direct comparisons to data in nonhuman animals. Filling this knowledge gap, by using comparative neuroimaging in humans and nonhuman animals conducting similar active tasks is highly challenging to achieve, but remains important because theoretical models of human cognition rely on information about neuronal mechanisms obtained in animal models (Corbetta et al. 2008; Reynolds and Heeger 2009; Duncan 2010; McLachlan and Wilson 2010; Zion Golumbic et al. 2012; Alho et al. 2014; Osmanski and Wang 2015). However, there is already indication that the assumption that attention similarly modulates brain processes in humans and primate models may not always hold (Patel et al. 2015; Malkinson and Bartolomo 2016).

Attention-related modulations in animal models have been most extensively studied in the visual domain (Herrero et al. 2008; Reynolds and Heeger 2009; Briggs et al. 2013; Caspari et al. 2015; Patel et al. 2015). Much less is known about the effects of auditory or audio–visual selective attention (Hocherman et al. 1976; Benson and Hienz 1978; Fritz et al. 2007; Lakatos et al. 2009, 2016; Niwa et al. 2015; Osmanski and Wang 2015). Direct cross-species comparisons between auditory attention-related modulations in humans and animal models are, to our knowledge, altogether absent. The results of neuroimaging studies in humans suggest that auditory attention can substantially modulate broad stretches of auditory cortex and beyond (Pugh et al. 1996; Grady et al. 1997; Petkov et al. 2004; Rinne et al. 2009; Woods et al. 2009; Da Costa et al. 2013; Alho et al. 2014). By comparison, in animal models, there is considerable diversity in the effects of active listening tasks on neuronal responses in primary or adjacent auditory cortical areas (Hocherman et al. 1976; Benson and Hienz 1978; Fritz et al. 2007; Atiani et al. 2009, 2014; Otazu et al. 2009; Niwa et al. 2015; Osmanski and Wang 2015). Given that behavioral training and task control are often more challenging to achieve in nonhuman animals than in humans, especially for auditory tasks (Selezneva et al. 2006; Fritz et al. 2007; Otazu et al. 2009; Atiani et al. 2014; Niwa et al. 2015; Osmanski and Wang 2015), lapses in task control could not only reduce but alter attention-related responses (Hocherman et al. 1976; Lakatos et al. 2016). Thus, evaluating the correspondences and divergences in attention-related modulations across species remains crucial for understanding how insights on the neurobiology of cognition obtained in animal models could extrapolate to humans.

In the present functional magnetic resonance imaging (fMRI) study, we mapped the large-scale effects of audio–visual selective attention in monkeys. For comparison, we also acquired fMRI data in humans performing identical tasks, focusing on the auditory modality for the cross-species comparisons. Two monkeys were trained to perform an auditory or visual task with identical bimodal stimuli. They were rewarded for attending to spatial changes in one sensory modality while ignoring those in the other modality. Our guiding hypothesis was that auditory attention would modulate a comparable territory of sensory areas in both species. In the monkeys, we observed a dichotomy of auditory and visual attention effects in specific cortical regions. Surprisingly, we found that a substantially more restricted set of auditory temporal cortical regions showed enhanced activations during the auditory task in the monkeys than in the humans. However, the results do not require appealing to an account based on evolutionary divergences in attention networks (Patel et al. 2015; Malkinson and Bartolomo 2016). Instead, lapses in selective attention in the monkeys appeared to have altered the pattern of attention-related modulations. Taken together, this study identifies the primate brain regions strongly modulated by attention-engaging auditory and visual tasks, compares auditory attention effects with those in humans, and raises the possibility that lability in performance on cognitive tasks in animal models can be at the source of a number of superficial cross-species differences in how attention modulates cortical networks.

## Materials and Methods

### Monkey Procedures

Two adult research-naïve male Rhesus macaques (*Macaca mulatta*) from a group colony of pair housed animals were involved in this study (M1 and M2). The pen sizes in our colony range from 130 × 240 cm to 215 × 240 cm. All are 230 cm high, and hatches between neighboring cages are used to increase the space available to the animals. The day/night cycle was natural and all tests were conducted during the hours of 8.00–18.00. Both animals were 3 years old at the beginning of the study and weighted 5 and 6 kg, respectively. When the study completed they were 5 years old and weighted 12 and 12.5 kg, respectively. All nonhuman animal work and procedures were performed at Newcastle University, UK and were approved by the Animal Welfare and Ethical Review Body at Newcastle University and by the UK Home Office. The work complies with the Animal Scientific Procedures Act (1986) and with the European Directive on the protection of animals used in research (2010/63/EU). We support the principles on reporting animal research stated in the consortium on Animal Research Reporting of In Vivo Experiments. All persons involved in animal handling and procedures were Home Office certified and the work was strictly regulated by the UK Home Office.

### Stimuli

The auditory stimulus was a macaque (“coo”) vocalization recorded from a male macaque that was unfamiliar to the 2 individuals tested. The coo vocalization was 400 ms in duration (including 8 ms onset and offset raised-cosine ramps). Left and right spatialized versions of the stimulus were created for headphone presentation during fMRI. This was done by playing the vocalization from a loudspeaker (Creative Inspire T10; distance 1 m from the macaque’s head; ±90° in azimuth; 65 dB SPL LAeq). The sound was recorded from within the macaque's ears using in-ear microphones (Knowles Electronics). During the sound recordings, the monkeys were seated in a primate chair with their head immobilized. This procedure resulted in strongly lateralized sounds (inter-aural level difference ca. 10 dB). When the sounds were first introduced, the monkeys systematically turned their eyes toward the sound sources left or right, verifying that they perceived the spatial lateralization of the sounds as expected. The 2 spatialized sounds were presented as left-left, right-right, and left-right pairs using a 200 ms (offset to onset) within-pair interval.

The visual stimulus was a picture of a monkey face (presented for a duration of 400 ms, subtended 5° visual angle). The background luminance of the screen was 81.63 cd/m^2^ and the Michelson contrast of the face image was 0.054. The picture was presented at the left or right of the visual screen, where the center of the picture was offset by ±5° from the center of the screen. As with the auditory stimuli, the pictures were presented as left-left, right-right, and left-right pairs with a 200 ms within-pair interval.

**Figure 1.**
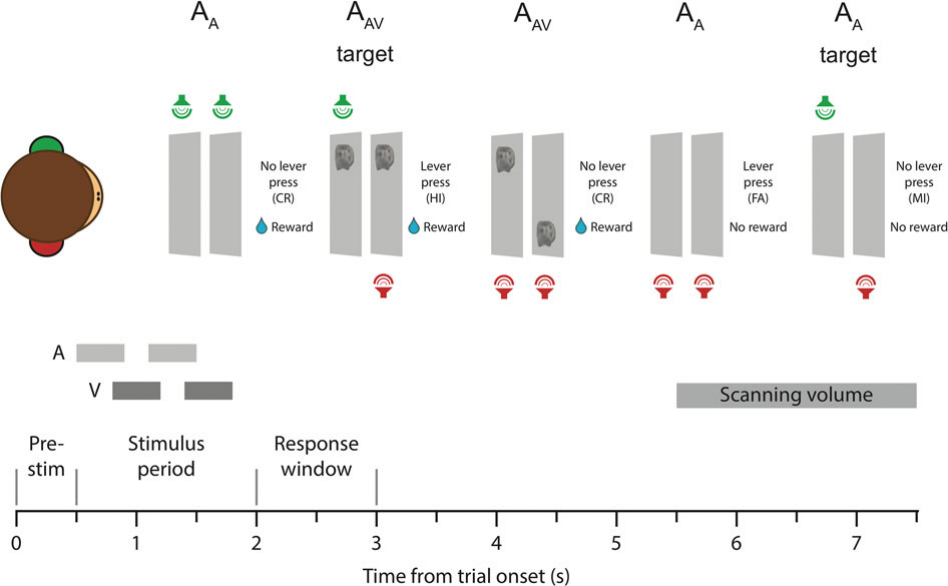
Auditory–visual selective attention task. (Top) Monkeys were presented with a monkey vocalization (a “coo” call) in left or right virtual acoustic space. The sounds were presented in left-left, left-right, or right-right pairs. In the illustrated auditory task, the monkeys were required to withhold pressing the response lever when the sounds of a pair appeared in the same location (left-left or right-right) and to press the lever to left-right target pairs to receive a juice reward. To help to cue the monkeys to the attended modality, most trials with stimuli (48% of all trials) were unimodal auditory (A_A_). The visual stimulus presented during bimodal (A_AV_) trials was a low-contrast grayscale monkey face displayed at the left or right of the visual screen and, in analogy to the auditory stimuli, the images were presented in left-left, left-right, or right-right pairs. Correct rewarded (CR or HI) and incorrect non-rewarded trials (FA or MI) are shown. Green, left ear. Red, right ear. (Bottom) The structure of the 7.5 s trial was identical in all stimulus and task conditions. In bimodal trials, the visual stimuli started 300 ms after the auditory stimuli. In unimodal trials, the stimuli were presented in the same positions as in bimodal trials. Juice rewards were presented immediately after a correct response in HI trials or at the end of the response window in a CR trial. In addition, a green screen was presented as a visual reward cue at the same time with a juice rewards (HI and CR trials). A red screen was shown immediately after an incorrect response or at the end of the response window for missed targets. The visual reward cues were shown until the end of the trial. The fMRI volume was acquired ~4 s after the offset of the second auditory stimulus, at the estimated peak of the hemodynamic response to sounds (Baumann et al. 2010).

The stimuli were presented during auditory (A) and visual (V) tasks in auditory only (A_A_), visual only (V_V_), or audio–visual (A_AV_, V_AV_) trials (Fig. 1). In the bimodal stimulation trials, the first part of the visual stimulus pair was always presented 300 ms after the onset of the first part of the auditory stimulus pair to avoid direct masking or competition between simultaneously presented auditory and visual stimuli. In A_A_ and V_V_ trials, the stimuli in the opposite modality were omitted. In these trials, the timing of the unimodal stimuli was identical to the stimuli of that modality in the bimodal trials. In bimodal trials, the auditory and visual pairs were randomly combined (i.e., the different auditory/visual combinations were as follows: A_LR_V_LR_, A_LL_V_LL_, A_RR_V_RR_, A_LR_V_LL_, A_LR_V_RR_, A_LL_V_LR_, A_LL_V_RR_, and A_RR_V_LL_). To help to cue the monkeys to the attended modality during bimodal trials, most of the trials with stimuli (48% of all trials) were unimodal (for details see section: Behavioral procedure during fMRI).

The monkey experiment was controlled using Cortex software (Salk Institute). The auditory stimuli were delivered using MRI-compatible headphones (NordicNeuroLab) at 65 dB SPL LAeq (calibrated with an NTI Audio XL2 sound level meter). Visual stimuli were presented on gray background. The visual stimuli were projected to a screen that the monkeys could see by way of a mirror in front of them. The scanner noise was attenuated with the ear cups around the headphones and foam around these (TempurPedic).

### Monkey Behavioral Training

The behavioral training proceeded in steps with increasing difficulty and complexity from initial lever press training toward the final scanner-ready task. This was not a linear process as occasionally it was necessary to return to the preceding training step with remedial training to restore performance. Following initial acclimation of the animals to laboratory testing, an MRI-compatible head post for head immobilization during MRI data collection was implanted under general anesthesia and aseptic conditions. After allowing sufficient time for a full recovery from the procedure (6 weeks), the animal was slowly acclimated with positive reinforcement to accommodate the necessary periods of head immobilization. We relied on operant training with preferred juice as reward to motivate the animals to correctly perform the task. During training and testing, the animals were on an individually customized fluid control protocol, which ensured that they stayed motivated, healthy and not overly thirsty. The animals had unrestricted access to fluid on days when they were not being trained and over the weekends.

Visual reward cues were used to supplement the juice reward delivery during correct trials (green screen) or to provide a cue to emphasize an incorrect trial which resulted in no reward (red screen). The juice reward and green screen were presented at the same time immediately after a correct lever press to targets or at the end of the response window (1000 ms, starting 200 ms after the end of the last stimulus) for correct nontarget trials. The red screen was shown immediately after an incorrect lever press or at the end of the response window for missed targets (see Fig. 1). The green and red screens were shown until the end of the trial. In addition to juice and visual rewards, we also used a time-out period as a feedback after incorrect trials during training.

The macaques were trained to press a lever to target stimulus pairs that contained a spatial location change and to withhold a response for nontarget stimulus pairs with no spatial change. Each trial was classified as a hit (HI; correct response to target), miss (MI; incorrect lack of response to target), false alarm (FA; incorrect response to nontarget), or correct rejection (CR; correct lack of response to nontarget; Fig. 1). HI rate, FA rate, and *d’* were calculated separately for each testing run (~100 testing trials). Performance was evaluated by monitoring *d’* [*d*′ = Z(hit rate) – Z(false alarm rate)] computed across a run and compared with chance level (Caporello and Gentner 2012; Caporello Bluvas and Gentner 2013). To define chance level *d’*, the relationship between responses given and the stimulus identity was permuted to simulate a monkey that gave the same responses but was oblivious to the stimulus conditions. A null distribution of *d’* values was created from 1000 permutations. Chance *d’* performance was defined as the 95% (one-tailed) point in the distribution. We proceeded to the next step in training when the monkeys stably performed at *d’* levels above chance for at least one testing run per testing day for at least 5 days.

Initially in training, we used consistent bimodal stimuli (i.e., A_LR_V_LR_, A_LL_V_LL_, and A_RR_V_RR_). Within 2 weeks of training, the monkeys learned to press the lever to targets and withhold responses to nontargets. However, when we then began auditory task training by reducing the contrast of the visual face stimulus, so that the animals would perform the task based on the sounds, we saw that the animals’ had fully relied on the visual stimuli and could not perform the task based on auditory stimuli. Auditory task training using only unimodal auditory stimuli proved to be challenging and it took several months for the monkeys to reach consistent performance. Once they were at criterion auditory task performance, we introduced the visual stimuli in 32% of the trials (all possible auditory–visual combinations). This ensured that there were a sufficient number of auditory only trials to remind the monkey that it was only being rewarded for correct performance on the auditory trials. Occasionally, during the introduction of bimodal stimuli, we presented a greater proportion of unimodal auditory trials at the beginning of the testing run to help the monkey to focus on the task. Otherwise, the unimodal and bimodal trials were presented in random order. The training procedure for the visual task was similar, starting with training on unimodal visual trials followed by introduction of bimodal trials. Once the animals were stably performing the auditory or visual task with bimodal stimuli they progressed to fMRI scanning (see next section).

During behavioral training, a number of parameters were adjusted automatically to ensure that the monkeys were under stimulus control. For example, if the monkey adopted a strategy of not responding (trying to get by on the rewards given for CRs) then we increased the proportion of target trials, the relative amount of juice reward to hit trials, and the time-out period for missed targets. If the monkey started responding to all trials (focusing on getting all the target trial rewards) then we increased the time-out delay for incorrect trials and the proportion of nontarget trials. However, it is important to note that task parameters were adjusted only during the behavioral training. During fMRI, all parameters were fixed and fully balanced across auditory and visual tasks (see next section).

We also acclimated the monkeys to the scanner chair, to scanner noise, and finally to performing the task in the scanner. After the fMRI data collection for the auditory task was completed, the animals were retrained to perform the visual task at above criterion levels for subsequent fMRI data acquisition. Depending on the monkey's motivation, a training session lasted 1–3.5 h resulting in 200–800 trials. Each animal performed more than 380 training sessions during 2 years of training.

### Behavioral Procedure During fMRI

During fMRI, each block of 10 trials had to be initiated by the macaque with a lever press to decrease the possibility of a free-running task where the animal does not participate at all but gets “free” juice rewards during nontarget trials. This lever press to start the block was not rewarded and was followed by a 600 ms wait period before the next block of trials commenced. Each trial started with a 500 ms pre-stimulus period. Next, the stimuli were presented during a 1500 ms stimulus presentation window. This was followed by a 1000 ms response window, 2500 ms delay, and a 2000 ms image acquisition period (see next section and Fig. 1). The acquisition of the fMRI volume started ~4 s after the offset of the auditory stimuli at the peak of the expected hemodynamic response to sounds (Baumann et al. 2010).

After a correct trial, juice reward and a green screen as a visual reward cue were provided immediately after the response in a HI trial or after the response window closed in a CR trial. After an incorrect trial, no reward was delivered and a red screen was shown in a FA trial or after the response window closed in a MI trial for the duration of the rest of the trial.

During fMRI, 20% of all trials contained no stimuli (baseline), 48% were unimodal trials within the cued modality (auditory only for the auditory task, visual only for the visual task), and the rest (32%) were bimodal trials. The presentation order of trials was randomized. All stimulus parameters were fixed throughout the fMRI data acquisition.

The first phase of fMRI data acquisition was conducted with the monkeys performing the auditory task. Once at least 15 scanning runs with criterion performance (above chance *d’*) were collected per animal, the animal was then switched to performing the visual task (requiring a few weeks of behavioral retraining). The experiment completed with the collection of data on the visual task. It was not possible for the animals to switch the tasks more regularly, as task switching required several weeks/months of retraining. Further, given the complexity and challenges of behavioral task training and fMRI during active tasks, we did not require that the animals fixate during stimulus presentation, using the infrared eye-tracker only to ensure that the animals were looking within the monitor screen during fMRI. Performance for the monkeys was more challenging in the scanner. We interspersed scanning sessions with lab-based training to improve performance as needed (see above section: Monkey behavioral training).

### Monkey fMRI Procedure

The animals were scanned in a primate dedicated vertical 4.7 Tesla MRI scanner (Bruker Biospec 47/60 VAS, GA-38 S gradient system; Bruker Medical). Functional images were acquired using a gradient-echo echo-planar sequence (GE-EPI; 7.5 s inter-volume time, volume acquisition time 2000 ms, echo time (TE) 22 ms, flip angle 90°, matrix 96 × 96, field of view (FOV) 9.6 × 9.6 cm^2^, slice thickness 2.0 mm with no gap, in-plane resolution 1 × 1 mm^2^, 20 axial slices covering most of the brain, bottom slice was aligned with the most inferior part of temporal lobes). In each trial, one functional volume was acquired after the stimulus presentation window closed (i.e., at the expected peak of the blood oxygen level-dependent response to stimulation; Fig. 1). This imaging paradigm allowed us to present the stimuli in silence and to avoid the effects of the loud scanner noise on brain activations (Petkov et al. 2008).

Two structural scans were acquired in each session aligned with the functional volumes. One of these was a full-head EPI with extra slices (28 total) which was used to help to register the functional volumes to the higher resolution anatomical image. The other image was an anatomical volume (MDEFT; TE 6 ms; repetition time (TR) 20 ms; matrix 192 × 192, FOV 9.6 × 9.6 cm^2^, slice thickness 2.0 mm with no gap, in-plane resolution 0.5 × 0.5 mm^2^) which had higher in-plane resolution.

Each scanning session occurred on a separate day. Overall, there were a total of 71 scanning sessions (39 auditory and 32 visual). A scanning session consisted of multiple runs of ~100 trials each (1–5 testing runs per scanning session depending on the animal's motivation). In total, there were 135 runs (83 auditory and 52 visual). Only the scanning runs that were completed with above criterion performance were submitted for fMRI analysis. In the auditory task, M1 completed 16 (from a total of 47) and M2 completed 16 (out of 36) scanning runs above criterion performance. In the visual task, M1 completed 18 (from a total of 29) and M2 completed 20 (out of 23) runs above criterion performance. These numbers do not include the training sessions that occurred without scanning (see above section).

### Monkey fMRI Data Analysis

The functional data of each scanning run were preprocessed and analyzed using FSL (version 5.0.8). The data were motion-corrected, high-pass filtered (100 s cutoff) and spatially smoothed (Gaussian kernel of 1 mm; full-width half maximum, FWHM). A general linear model with 9 explanatory variables (unimodal and bimodal HI, MI, CR and FA trials, and the duration of inter-image interval) was defined. The duration of inter-image interval was used as a variable of no interest, in order to model the effects of between-block inter-image variation on the signal magnitude (each block of 10 trials was initiated by the monkey). Functional data of each scanning run were coregistered (using FSL’s FLIRT, linear affine transformation) via the intermediate anatomical scans to a template monkey brain (McLaren et al. 2009; Petkov et al. 2015) that is in register with a macaque brain atlas in stereotactic coordinates (Saleem and Logothetis 2012).

Higher level analysis was conducted across runs and animals. Using FreeSurfer tools (version 5.3, www.freesurfer.net), the contrast parameter estimates from the first-level analysis (within a run) were resampled to the cortical surface of the template monkey brain (McLaren et al. 2009; Petkov et al. 2015) and smoothed on the surface (5 mm FWHM). Higher level analysis was conducted in surface space using FSL’s Permutation Analysis of Linear Models (PALM; version alpha35, Winkler et al. 2014; 5000 permutations) using cluster-mass correction as specified in the results. The tables in Figure Supplements report anatomical structures (Saleem and Logothetis 2012) within each significant cluster and the coordinates of the maximum Welch’s *v*-stat values in that anatomical structure.

For the Region of Interest (ROI) analysis, 4 anatomical ROIs were defined on the inflated cortical surface subdividing the macaque superior temporal gyrus (STG) into 4 segments in the anterior–posterior direction. ROI mean signal magnitudes were computed separately for each ROI and hemisphere. For analysis, the ROI means were collapsed across hemispheres. Mixed ANOVAs were used to investigate the main effects of ROI (1–4) and ROI × task (A, V) interaction. The degrees of freedom were Greenhouse–Geisser corrected when Mauchly’s test indicated that the assumption of sphericity did not hold. The original degrees of freedom are reported together with the corrected *P-*value and the correction term ε. Levene’s test was used to test for equality of error variances. Further, the difference between auditory and visual tasks in each ROI was analyzed using unpaired *t*-tests in FSL’s PALM. Significance was assessed using permutation (*n* = 5000) inference, correcting for multiple comparisons.

### Human Subjects

Fourteen humans (age 20–39 years, mean 25.8 years; 11 female) participated in the human component of this study conducted at the Advanced Magnetic Imaging Centre, Aalto University, Finland. All subjects self-reported on a questionnaire as being right-handed, having normal hearing, normal or corrected-to-normal vision, and no history of psychiatric or neurological illnesses. Informed written consent was obtained from each subject before the experiment. The human experimental protocol was approved by the University of Helsinki Ethical Review Board in the Humanities and Social and Behavioural Sciences.

### Stimuli and Human Behavioral Procedure

Before the fMRI session, subjects were trained to perform the tasks in one 30–45 min training session, which occurred 2–3 days prior to scanning. The auditory and visual stimuli were identical to those used in the monkey study except that, in the human study, the spatialized sounds were created using the pan function of Audacity (1.3.14-beta, http://sourceforge.net/projects/audacity) and only bimodal stimulus conditions were used (A_AV_ and V_AV_).

The sounds and pictures were presented in 20 s blocks alternating with 13 s breaks. There were 3 auditory and 2 visual conditions (16 blocks for each of the 3 auditory conditions and 8 blocks for each of the 2 visual conditions). Here only the data from the auditory and visual conditions with similar stimuli and tasks as in the monkey study are reported. As no feedback was given, the inter-trial interval was shorter (800–1500 ms) than in the monkey study. That is, in the human study, we used a block design (fMRI analysis was based on effects over several trials in a block, whereas in the monkey study activations to each trial were analyzed separately). The 13 s breaks in between the task blocks consisted of a rest period of 10 s with no stimuli followed by an instruction period of 3 s. During the instruction period, a graphic symbol indicated the next task. Subjects responded to targets by pressing a button with their right index finger. Continuous scanning with jittered data acquisition (i.e., stimulus presentation and data acquisition were not time locked) was used.

The human experiment was controlled using Presentation software (Neurobehavioral Systems). The auditory stimuli were delivered using Sensimetrics S14 insert earphones (Sensimetrics Corporation) at a comfortable listening level, adjusted individually for each subject. Visual stimuli, instruction symbols and a fixation cross in the middle of the screen were presented on a gray background. The visual stimuli were projected onto a mirror fixed to the head coil so that they could be seen by the subjects. The scanner noise was attenuated by the insert earphones, circum-aural ear protectors (Bilsom Mach 1, Bacou-Dalloz Inc.), and viscous foam pads attached to the sides of the head coil.

### Human fMRI Data Acquisition and Analysis

Functional brain imaging was carried out with a 3 Tesla MAGNETOM Skyra (Siemens Healthcare). In the beginning of each session, a high-resolution anatomical image (slice thickness 1.0 mm, in-plane resolution 1 × 1 mm^2^) was acquired. Functional images were acquired using a GE-EPI sequence (TR 2220 ms, TE 30 ms, flip angle 78°, matrix 96 × 96, FOV 18.9 × 18.9 cm^2^, slice thickness 2.0 mm with no gap, in-plane resolution 2 × 2 mm^2^, number of slices 27). The middle EPI slices were aligned with the Sylvian fissure of each subject based on their high-resolution anatomical image. The imaging area covered the superior temporal lobe, insula, and most of the inferior parietal lobe in both hemispheres (shadowed area in Fig. 4). The functional scanning was divided in two 18 min runs with 477 functional images each.

FreeSurfer (version 5.3, www.freesurfer.net) was used for reconstruction of cortical surfaces and coregistration (bbregister, boundary-based registration). Functional data was motion-corrected, resampled to the standard cortical surface, and surface-smoothed (10 mm FWHM). First, the data of each run were separately analyzed in surface space and then a second-level analysis (fixed effects) was conducted to combine the data from the 2 runs. Finally, group analysis was performed using the PALM tool of FSL, as with the monkey data analyses.

## Results

### Monkey Performance During fMRI

Over the course of 2 years, 2 monkeys (M1 and M2) were trained and rewarded for detecting either auditory or visual targets during unimodal or bimodal stimulation trials (Fig. 1). To help to cue the monkeys to the attended modality, most stimulation trials were unimodal. The auditory stimulus was a monkey vocalization (a “coo” call) presented in left or right virtual acoustic space. The sounds were presented in left-left, right-right, or left-right pairs (see Materials and Methods for details). The visual stimulus was a low-contrast grayscale monkey face image displayed at the left or right of the visual screen and, in analogy to the auditory stimuli, the images were presented in left-left, right-right, or left-right pairs. The monkeys were trained to make a lever press when the stimulus pair in the rewarded modality contained a spatial change (i.e., target left-right pair) and withhold pressing the lever when the stimulus pair in the rewarded modality appeared in the same location (i.e., nontarget left-left or right-right pairs). Pressing the lever to a target (HI) and not pressing the lever to a nontarget (CR) were rewarded as correct responses. Incorrect responses (FA; MI) were unrewarded.

**Figure 2.**
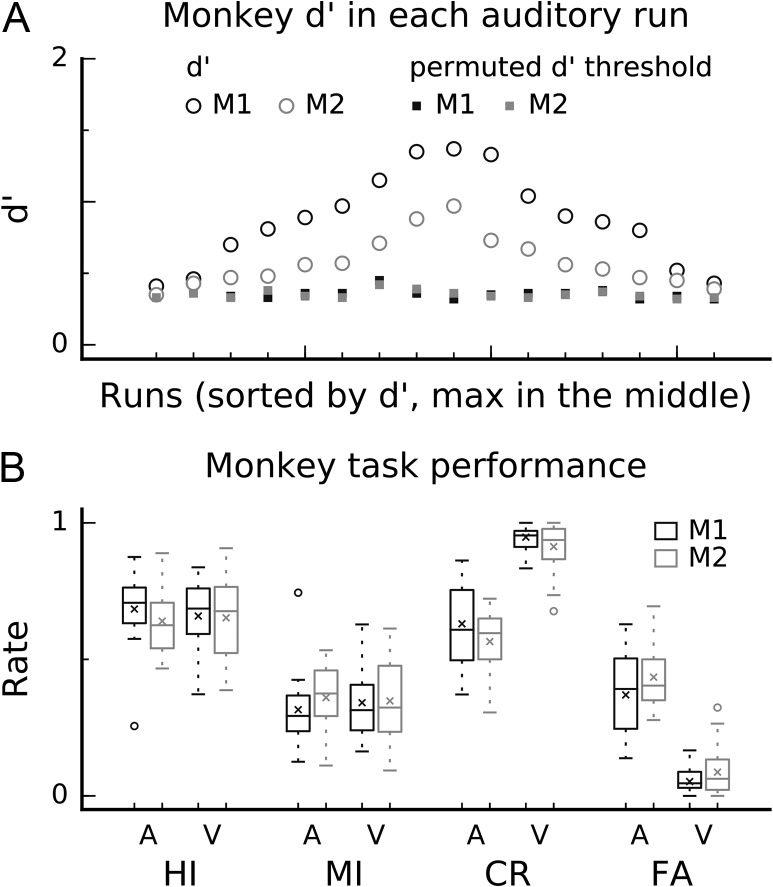
Monkey and human performance during fMRI. (*A*) Top row shows *d’* (circles) and permuted *d’* threshold (squares) values for the 2 monkeys (M1 black, M2 gray) in each auditory task above-threshold run. The runs are sorted so that highest *d’* values are in the middle. (*B*) Bottom row shows HI, MI, CR, and FA rate for the 2 monkeys in the auditory and visual task. The whiskers indicate 1.5 times the interquartile range (IQR) from the first and third quartile and × shows the sample mean.

We aimed for each monkey to provide at least 15 fMRI runs (each with ca. 100 trials) per task with above criterion (*d’* levels above chance, see Fig. 2*A* and Materials and Methods: Monkey behavioral training). In M1, we obtained 16 and 18 above criterion fMRI runs in the auditory and visual task, respectively. M2 completed 16 auditory and 20 visual runs with above criterion performance. Initially, we aimed at fully matching auditory and visual task performance (e.g., during training using distinct auditory stimulus differences and low-contrast pictures). However, this proved impossible in practice because although we were able to match the monkeys’ HI and MI rates across the tasks, there was a benefit for nontarget trials in the visual modality that could not be matched without affecting HI and MI rates (Fig. 2*B*). Thus, overall performance was more accurate in the visual (mean *d’* = 2.1) than auditory (mean *d’* = 0.7) task (permutation inference using unpaired *t*-test, 2 sided, 5000 permutations, *P* = 0.0002). Nonetheless, the monkey fMRI auditory-attention related results that we report next are consistent when analyzing trials in which performance across the modalities is matched (HI) or mismatched (CR).

### Monkey fMRI: Attention-Related Modulations

**Figure 3.**
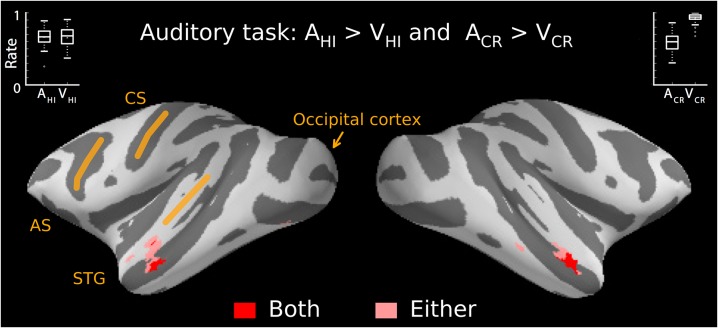
Auditory attention-related modulations in monkeys. Areas showing stronger activations during auditory than visual task performance (A_AV_ > V_AV_) during HI and CR trials. Results are shown on the left and right hemisphere inflated cortical surfaces (gyri: light gray; sulci: dark gray). Colors show areas where both (red) or either (pink) comparisons showed significant effects. Note the overlapping activation enhancements in both HI (matching A and V performance, top left) and CR (V better than A, top right) contrasts. The comparisons were performed in surface space and permutation inference (using Welch’s *v* test) was used to assess statistical significance (2 monkeys, comparison across 32 auditory task > baseline and 38 visual task > baseline first-level contrast parameter estimates, the runs of one monkey were treated as a permutation and variance group to accommodate heteroscedasticity, initial cluster-forming *Z* threshold 3.1, cluster-corrected *P* < 0.05). Orange lines and text mark key anatomical landmarks. Abbreviations: CS, central sulcus; AS, arcuate sulcus; STG, superior temporal gyrus. For a summary of anatomical regions, see Fig. 3—Supplement 1. Figure 3—Supplements 2–5 show visual attention-related modulations (V_AV_ > A_AV_), eye-movement data, and stimulus-dependent activations to sounds.

Analogous to previous human neuroimaging studies (e.g., Petkov et al. 2004; Rinne 2010), we evaluated auditory attention-related modulations by comparing activations during auditory (A_AV_) versus visual (V_AV_) tasks across conditions with identical bimodal stimuli. We first contrasted activations during auditory and visual HI trials (with lever presses) where performance (HI rate) was well matched across modalities (Fig. 3). In addition, we also compared activations during CR trials (no lever presses), where performance (CR rate) was better during the visual than auditory task. Note that these comparisons were made across auditory and visual trials with identical stimuli, performance, rewards, and motor responses, with the only difference being the task that was performed. During the auditory task, significant (cluster corrected *P* < 0.05) activation enhancements were prominent in the anterior STG and superior temporal sulcus (STS), which overlapped for both comparisons (HI and CR; red regions in Fig. 3; for a summary of anatomical areas, see Fig. 3—Supplement 1). By comparison, performance on the visual task (contrasting V_AV_ vs. A_AV_) was associated with significantly enhanced activations in the occipital cortex, STS, parietal cortex, and frontal cortex (Fig. 3—Supplement 2 and 3). The variability in eye looking position during the auditory and visual tasks was not statistically different (repeated-measures ANOVA, *F*_1,91_ = 2.3, *P* = 0.133; Fig. 3—Supplement 4). Thus, any effects associated with eye movements cannot easily explain the activation differences observed during auditory and visual tasks.

### Monkey fMRI: Stimulus-Dependent Activations

Activations to sounds were extracted by contrasting activations during the visual task with bimodal (V_AV_) versus unimodal (V_V_) stimulation (Petkov et al. 2004). As expected, stimulus-dependent activation enhancements in response to the sound stimulation were seen in and around auditory cortex (Fig. 3—Supplement 5). These activations were only observed at a more liberal (nonsignificant) statistical threshold (cluster-forming *Z* threshold 2.3, cluster corrected *P* < 0.15). In the visual modality, no activation clusters surviving the cluster-forming *Z* threshold 2.3 were detected, possibly because of the use of a low-contrast stimulus. Thus, stimulus-dependent activations were weak, underscoring the substantially stronger and broader modulations when the monkeys conduct active tasks (Fig. 3).

### Human Performance and fMRI Attention-Related Modulations

**Figure 4.**
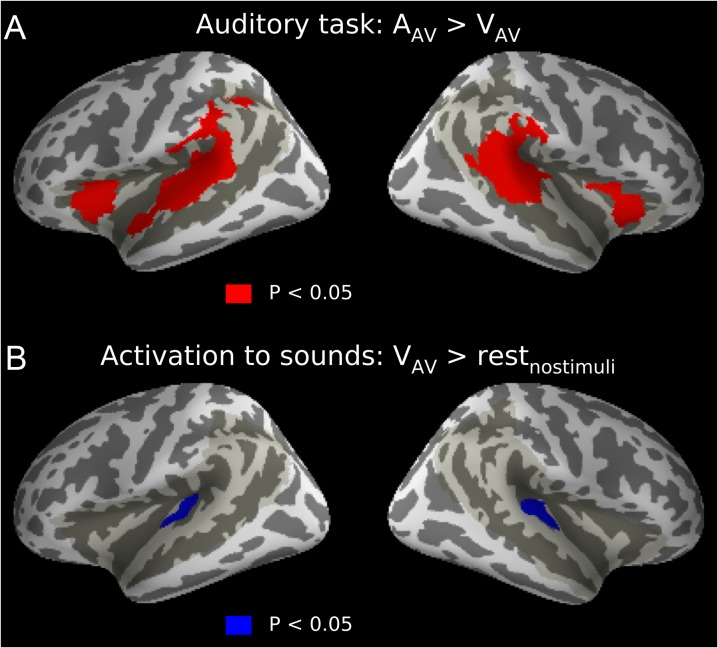
Auditory attention-related and stimulus-dependent effects in humans performing the monkey tasks. (*A*) Auditory attention-related modulations were evaluated by comparing activations to identical bimodal stimulus blocks presented during the auditory and visual tasks (A_AV_ > V_AV_; mean effect across 14 lower level contrast parameter estimates, permutation inference using *t* stat, threshold-free cluster enhancement, corrected *P* < 0.05). For a summary of anatomical regions, see Fig. 4—Supplement 1. (*B*) Auditory stimulus-dependent modulations were extracted by comparing activations to sounds presented during the visual task (no directed auditory attention) in relation to rest (no stimuli); initial cluster-forming *Z* threshold 2.3, cluster-corrected *P* < 0.05. Shadowed region shows the imaged area.

We also acquired human fMRI data while the human subjects performed similar tasks as the monkeys and with the same bimodal stimulation conditions. Unsurprisingly, humans performed these tasks designed for monkeys with high accuracy and made few errors. Task performance (*d’*) was better during the visual (mean *d’* = 4.2) than auditory (mean *d’* = 3.6) task (permutation inference using paired *t*-test, 2 sided, 5000 permutations, *P* = 0.0002), an effect also seen with monkey performance during fMRI. The human fMRI focused on areas around the Sylvian fissure (i.e., auditory cortex and adjacent regions; shadowed area in Fig. 4) and only bimodal stimulation conditions were used (A_AV_ and V_AV_), since the unimodal conditions that served as cues for the monkeys were not required. We found significantly stronger activations (threshold-free cluster enhancement, corrected *P* < 0.05) during the auditory than visual task in bilateral regions of middle and posterior STG, inferior parietal lobule and frontal operculum (FO) as well as in left anterior STG (Fig. 4*A*). Auditory stimulus-dependent activations were measured by comparing activations to sounds presented during the visual task (attention directed to the visual stimuli) with the activations during the rest periods with no stimuli (Rinne et al. 2009). As expected, this contrast revealed stimulus-dependent activations (cluster-forming *Z* threshold *Z* > 2.3, cluster corrected *P* < 0.05) in more restricted areas around the Heschl’s gyrus bilaterally, in or near human primary auditory cortex (Fig. 4*B*).

### Monkey fMRI: ROI Analysis of STG Activations

**Figure 5.**
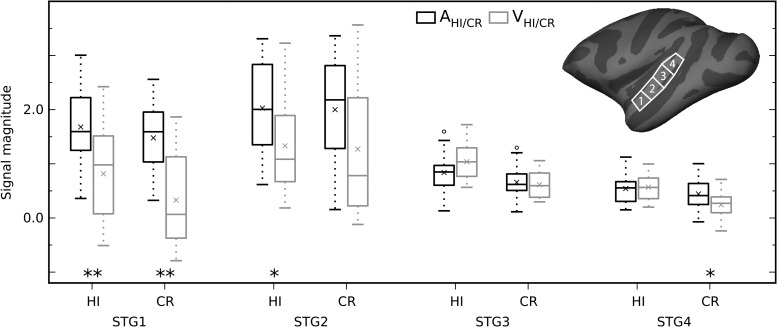
ROI analysis of attention-related modulations in monkey STG. The box plots show mean signal magnitudes (2 monkeys; to remove outliers, the central 80% of values were included in each case leaving 24 auditory and 30 visual runs) in each anatomically defined STG ROI (collapsed across hemispheres) for correct bimodal trials during the auditory (black) and visual (gray) task (× shows the sample mean). The insert at top right shows the location of the left hemisphere ROIs. Task-related modulations were not uniform across the STG ROIs (significant main effect of task and task × ROI interaction for both HI and CR trials, see Results). Asterisks indicate significantly higher signal magnitude during auditory than visual tasks (permutation-based significance testing using unpaired *t*-tests, one-sided, 5000 permutations, multiple comparisons corrected, **P < 0.01, *P < 0.05, significant *P* values in STG1 = 0.0008 and 0.0002; STG2 = 0.0344; STG4 = 0.048). Middle STG regions (STG3) showed no significant effects. Signal magnitude is in relation to no-stimulus baseline trials.

Based on the present human results, which largely recapitulate prior human reports of regions modulated by auditory attention (Pugh et al. 1996; Petkov et al. 2004; Rinne et al. 2009; Woods et al. 2009), we were surprised that the contrasts between the auditory and visual task in monkeys revealed minimal effects in middle and posterior auditory STG regions (compare Figs 3 and 4*A*). To further investigate activations throughout STG, we conducted a ROI analysis in 4 anatomically subdivided STG regions. The signal magnitudes in the STG ROIs showed that the pattern of task-related modulations was not homogenous throughout auditory cortical areas along the STG (Fig. 5). This was confirmed by mixed ANOVAs with factors ROI (1–4) and task (A, V) conducted separately for correct trials (HI and CR). The results showed a significant main effect of task (HI: *F*_1,52_ = 11.8, *P* = 0.0012; CR: *F*_1,52_ = 20.7, *P* = 0.00003) and ROI × task interaction in both cases (HI: *F*_3,156_ = 9.88, *P* = 0.00007, *ε* = 0.724; CR: *F*_3,156_ = 6.97, *P* = 0.00154, *ε* = 0.655; Levene’s test of equality of error variances, *F*_1,52_ < 2.5, *P* > 0.1 for each ROI and both ANOVAs). These effects resulted from the significantly higher signal magnitudes during the auditory than visual task in the 2 most anterior ROIs for HI trials and in the most anterior and posterior ROIs for CR trials (permutation inference using unpaired *t*-test, one-sided, 5000 permutations, multiple comparisons corrected; Fig. 5). Notably, auditory selective attention effects were absent in the middle STG ROI (STG3) including primary auditory cortex. These results suggest that systematic attention-related modulations in middle-posterior STG regions were not absent in the A_AV_ > V_AV_ comparisons (Fig. 3) simply because of lower statistical power.

### Monkey fMRI: Task General Modulations (Comparing Correct and Incorrect Trials)

**Figure 6.**
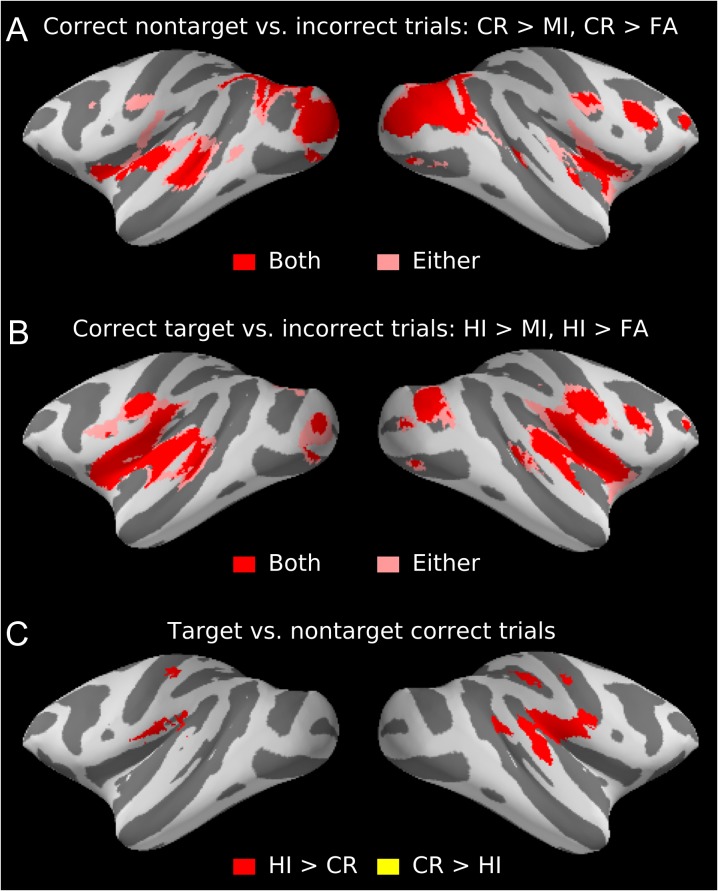
Enhanced activations during correct versus incorrect trials. Shown are the results of comparisons correct > incorrect trials in monkeys collapsed across auditory and visual tasks (permutation using Welch’s *v* test, one-sided, 2 monkeys, comparison across 70 vs. 70 first-level contrast parameter estimates, the runs of one monkey were treated as a permutation and variance group to accommodate heteroscedasticity, cluster corrected *P* < 0.05). (*A*) Activation enhancements for correct nontarget trials relative to incorrect MI or FA trials. Colors show areas where both (red) or either (pink) comparisons showed significant effects. For anatomical regions, see Fig. 6—Supplement 1. (*B*) Activation enhancements for correct target trials relative to incorrect trials (see Fig. 6—Supplement 2). (*C*) Comparisons between correct target (HI) and nontarget (CR) trials (Fig. 6—Supplement 3). Initial cluster-forming *Z* threshold was 5.5, 5.5, and 4.2 in *A*, *B*, and *C*, respectively.

**Figure 7.**
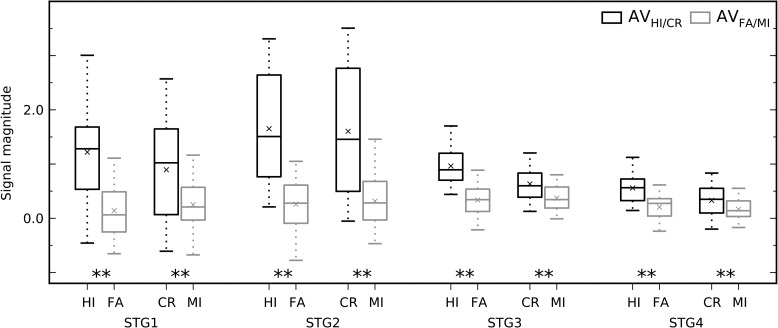
Performance during both tasks strongly modulates the monkey STG. The box plots show mean signal magnitudes in each STG ROI (collapsed across hemispheres and tasks) for correct trials (HI/CR; black) and incorrect trials (FA/MI; gray). To remove outliers, the central 80% of values were included, leaving a sample size of 56 in each case. Signal magnitudes were strongly enhanced in correct trials in all STG ROIs (compare with Fig. 5). Note the enhanced activations during correct trials in both HI versus FA (response given) and CR versus MI (no response) comparisons. Analyses as for Fig. 5. Multiple-comparison corrected *P* values from left to right: 0.0002, 0.0004, 0.0002, 0.0002, 0.0002, 0.0002, 0.0002, and 0.0026; **P < 0.01.

**Figure 8.**
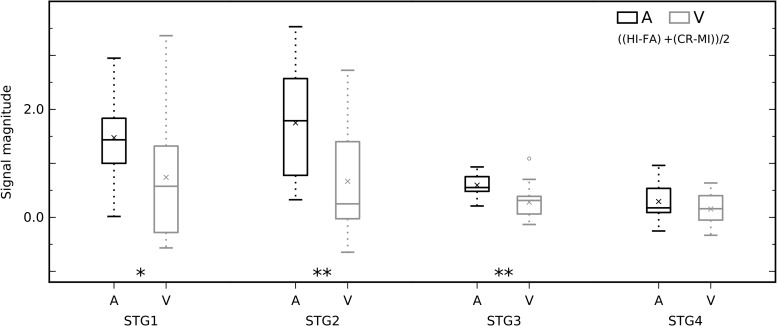
The activation difference between correct and incorrect trials in STG is greater during auditory than visual task. The box plots show the effects of auditory and visual task performance (difference between correct and incorrect trials) in each ROI. Similar to the results for Fig. 5, STG1 and STG2 ROIs show significant activation enhancement during the auditory task. Importantly, this comparison reveals a strong effect also in STG3. Analyses as for manuscript Fig. 5. Multiple-comparison corrected (significant) *P* values from left to right: 0.0116, 0.0008, and 0.0002; **P < 0.01, *P < 0.05.

Next, we evaluated whether and how performance on either task modulates monkey brain activations. Analysis of activations during correct trials versus incorrect trials (collapsed across tasks) revealed significantly enhanced activations in a substantial set of bilateral areas, involving hierarchically early auditory and visual cortical areas and fronto-temporal regions around the lateral sulcus (Fig. 6*A*, *B*; Fig. 7 show signal magnitudes in the 4 STG ROIs). Motor response-related activations cannot easily explain these effects observed in wide regions, because the results were largely similar regardless of whether a lever press was withheld (CR > MI; Fig. 6*A*) or given (HI > MI; Fig. 6*B*). The direct comparison between HI and CR trials showed enhanced activations mainly in regions around the central sulcus that are likely to be related to motor execution of level presses to targets (Fig. 6*C*). Activation enhancements during CR and HI trials around the central sulcus could well be related to sensations and movements associated with the juice reward (Patel et al. 2015). Thus, the contribution of sensorimotor effects (i.e., related to lever pressing and juice reward) on the enhanced activations observed during correct trials appears to be small or negligible. Taken together these results revealed substantial task-general modulations of activations in wide regions of STG and FO during task performance, regions which, by contrast, in humans showed strong auditory attention-related modulation in the auditory versus visual task comparisons (Fig. 4*A*). Importantly, when performance was taken into account, by comparing the difference between correct and incorrect trials in auditory and visual tasks, also more posterior STG regions showed enhanced activations during the auditory task (Fig. 8). That is, although STG3 ROI showed no activation differences between auditory and visual correct trials (Fig. 5), activations in that ROI are modulated significantly more by auditory than visual task performance.

## Discussion

The present fMRI study was designed to investigate attention-related modulations in the monkey brain by comparing brain activations to identical stimuli presented during an auditory or visual task. After 2 years of behavioral training, monkeys performed the tasks during fMRI above criterion (*d’* higher than chance, see Materials and Methods). Training the monkeys to conduct the selective attention task in the noisy scanner was very challenging and required several training steps during which the monkeys were required to perform the task to criterion performance before progressing. Ultimately the resulting performance levels are comparable to other macaque reports using challenging visual or auditory tasks (e.g., Messinger et al. 2001; Lakatos et al. 2016).

The auditory and visual tasks were associated with systematic activation enhancements in specific sensory cortical regions and in fronto-parietal areas. These findings provide initial insights into how attention-engaging auditory and visual tasks modulate large-scale activations in the primate brain. Surprisingly, however, auditory attention-related effects in monkeys were mainly observed in regions of the anterior superior temporal lobe, whereas in human subjects performing the same tasks as the monkeys a much more extensive set of superior temporal and other regions showed activation enhancements during auditory attention. Further analyses ruled out that this was a trivial discrepancy because of subthreshold effects in monkeys. Instead, we found task-general modulations in broader supratemporal regions during *both* the auditory and visual tasks. The combined set of regions in the monkeys susceptible to modulations during active tasks is remarkably similar to the regions showing auditory attention enhancements in humans. Given that behavioral performance measures alone cannot ensure a match in cognitive state in humans and monkeys, whether or not behavioral measures are matched, these observations are of considerable importance for identifying cross-species correspondences or sources of divergence in how attention modulates cortical regions.

### Correspondences in Auditory Attention Effects in Humans and Monkeys

In previous human fMRI studies on auditory attention, attention-related modulations are identified as the difference between activation to sounds presented during attended and ignored conditions with identical stimuli (e.g., Petkov et al. 2004; Rinne 2010; Alho et al. 2014). Such an approach allows examining activation modulations associated with the direction of attention in the absence of stimulus-dependent differences. Similarly, in the present study, we contrasted activations during identical bimodal stimulation conditions (A_AV_ > V_AV_) to investigate auditory attention-related modulations in monkeys. We also compared the results with those in humans performing similar tasks. In monkeys, the contrast A_AV_ > V_AV_ computed across trials with correct performance (HI and CR) revealed significant clusters showing attention-related modulations mainly in the anterior temporal cortex (Fig. 3). In humans, the areas modulated by auditory attention (although showing much more extensive activation enhancement throughout STG) also showed involvement of the anterior temporal cortex (Fig. 4*A*). To our knowledge, these monkey fMRI results show the first systematic modulation of regional cortical responses by an auditory attention-engaging task in nonhuman animals, with a certain level of correspondence in relation to the results in humans.

The effects in the anterior superior temporal lobe regions during the present auditory task involving spatial stimulus changes may be surprising. The ventral processing stream, which includes anterior STG, is thought to process non-spatial auditory object features (Rauschecker 1998; Rauschecker and Scott 2009), whereas spatial features are processed in more posterior regions within the dorsal stream. Consistent with this dual-stream model (Rauschecker and Scott 2009), previous human fMRI studies have shown stimulus-dependent sensitivity to pitch and location in, respectively, anterior and middle/posterior STG regions (Griffiths et al. 1996; Arnott et al. 2004; Bendor and Wang 2006; Alho et al. 2014; Häkkinen et al. 2015). However, activations in anterior and posterior regions of human STG also depend on the characteristics of the listening task. Auditory discrimination tasks are systematically associated with activation enhancements in anterior and middle STG regions, while *n*-back memory tasks performed on the same types of stimuli result in activation enhancements in more posterior STG and inferior parietal regions (Rinne et al. 2009, 2012; Häkkinen et al. 2015). Such task-dependent activation patterns are similarly observed irrespective of whether the tasks are performed on pitch-varying, spatially-varying, or communication (i.e., vowels) sounds (Harinen and Rinne 2013; Häkkinen et al. 2015). Thus, stimulus-specific processing does not appear to strongly contribute to the activation observed during active listening tasks (Häkkinen et al. 2015). It is noteworthy that, although anterior STG regions have been implicated in voice processing (von Kriegstein et al. 2003; Belin et al. 2011; Perrodin et al. 2015), the present enhanced activations in anterior STG in both monkeys and humans cannot be easily explained by voice- or sound-quality specific processing of the “coo” sound. Unlike previous studies on voice processing, in the present study, only one “coo” sound was repeatedly presented in all conditions. This is likely to result in reduced, rather than enhanced, voice-related activity (Belin and Zatorre 2003; Petkov et al. 2008). Further, any activation in anterior STG related to voice processing would have been present also in the sound versus silence comparison (Fig. 4*B*; Fig. 3—Supplement 5), which was not the case. Thus, based on the prior human fMRI results during active listening, we suggest that the present enhanced activations in anterior STG in monkeys and humans are related to the specific requirements of the auditory discrimination task, an interpretation that finds support in the broader auditory and visual literature (Karnath 2001; Häkkinen et al. 2015).

### Results in Relation to Models of Attention

Although the present study focused on investigating auditory attention effects, we also observed significant activity enhancements in occipital, inferior temporal (STS), dorsal parietal and frontal areas while monkeys performed the visual task. Generally, these areas showing visual attention effects are in line with those reported in previous monkey fMRI work on visual attention networks (Caspari et al. 2015; Patel et al. 2015). The frontal and parietal activations during visual attention task are consistent with current theoretical models of goal-directed behavior, as these regions belong to the so-called dorsal attention network (Corbetta and Shulman 2002; Corbetta et al. 2008). We also found that certain dorsal parietal and frontal regions showed enhanced activations associated with correct performance on both auditory and visual tasks (Fig. 6*A*,*B*). This suggests an overlap in dorsal parietal and frontal areas associated with auditory and visual task performance, which, to our knowledge, has previously only been reported in human fMRI studies using auditory and visual tasks (Shomstein and Yantis 2006; Salmi et al. 2007; Braga et al. 2016). Some of these parietal and frontal regions also seem to overlap with those that form the multiple demand network, which is flexibly engaged in behavioral control and selection across different forms of cognitive demands or sensory inputs (Duncan 2010).

However, the prevailing theoretical models of attention and goal-directed behavior focus on parietal and frontal attention networks. Auditory models, in turn, focus mainly on stimulus-specific processing. These models are not able to predict the strong activation modulations that we observed in wide auditory regions in the human temporal cortex during active listening. Our comparative fMRI study on audio–visual selective attention bridges work on attentional selection involving higher order brain networks and studies on selective attention effects on sensory processes, which provides a basis for updating or revising current models. Moreover, the results identify effects in a number of neurophysiologically understudied regions that are ripe for empirical pursuit using selective attention tasks.

### Cross-Species Divergences in Auditory Attentional Modulations?

Direct comparisons between activations during auditory and visual tasks with identical stimuli revealed no significantly enhanced activations in middle-posterior STG regions during auditory attention in monkeys. Further, the ROI analysis indicated that the lack of effects in these regions was not simply due to lower statistical power (STG3 ROI in Fig. 5). This was surprising as human studies, including the present one, systematically report strong attention-related modulations across a variety of auditory task or stimulus conditions particularly in middle-posterior STG regions (Pugh et al. 1996; Grady et al. 1997; Petkov et al. 2004; Rinne et al. 2009; Woods et al. 2009; Da Costa et al. 2013; Alho et al. 2014). Given these differential results in monkeys and humans, is it possible that the results can be explained by attention differentially modulating cortical networks in humans and monkeys (evolutionary divergence account)?

Additional analysis of the monkey data showed that activations in wide STG regions were modulated by performance during both auditory and visual conditions (task general modulations contrasting correct > incorrect trials; Figs 6 and 7). This pattern of results could occur if, at least on some trials, the monkeys had lapses in selective attention and were not fully able to ignore the stimuli in the unrewarded (to-be-ignored) modality (Lakatos et al. 2016). In other words, the monkeys may have conducted a more or less bimodal task on some trials. Any attention to sounds during the visual task would effectively diminish auditory attention-related effects (in A > V comparisons), especially in regions such as middle STG known to be strongly modulated by auditory attention in humans (Petkov et al. 2004; Rinne et al. 2009; Woods et al. 2009; Da Costa et al. 2013; Salo et al. 2013; Alho et al. 2014). Intriguingly, areas in anterior STG (where significant A > V differences were observed) appear to be less susceptible to such influences. Possibly, as noted above, these anterior STG effects are driven by the specific requirements of the auditory task. By contrast, attention-related differences in middle-posterior STG regions may be more susceptible to being masked or “lost” (in A > V comparisons) during labile selective-attention performance.

When the fMRI performance effects are taken into account (comparing the difference between correct and incorrect trials in auditory and visual tasks), significantly enhanced activations associated with auditory task performance were revealed in wider STG regions extending from anterior (STG1 and STG2 ROIs) to middle STG (STG3; Fig. 8). This pattern of activation modulations more closely matches that observed in humans during auditory-selective attention suggesting that activations in auditory cortex are similarly modulated by attention-engaging tasks in both monkeys and humans. Thereby, our results do not require appealing to evolutionary divergences in how attention modulates cortical networks, since in the monkeys lapses in selective attention appear to have diverted auditory attention effects in certain cortical regions. It is important to note that, in addition to the comparisons between correct and incorrect trials (Figs 6 and 7), which however could be affected by a number of performance related factors, this interpretation is supported by the comparisons between auditory and visual tasks with matching conditions (Fig. 8).

### Implications for Identifying Correspondences Across the Species in Studies of Attention

The results discussed above raise the possibility that, even when the experimental conditions and stimuli are similar, task performance strategies can be at the source of a number of surface differences in how attention modulates activations in monkeys and humans. In the present study, the accuracy of monkey and human performance (*d’*) during fMRI were unmatched: Humans performed the tasks designed for monkeys using the same stimulation conditions and task requirements during fMRI. However, matching behavioral performance across species can be extremely difficult to achieve and may not help because task strategies and associated brain modulations could differ even under identical accuracy. For instance, high accuracy in task performance could indicate either well-focused attention or an overly simple task with low cognitive load. An easy task could be associated with labile attentional focus and cognitive status. Low accuracy, in turn, could indicate well-focused attention on a highly demanding task or that the task is too easy and not motivating. That is, while accuracy measures are important and useful indices of task performance, matched task accuracy across species does not guarantee that the monkeys and humans are using identical behavioral strategies to perform the tasks and are in an identical cognitive state. Thus, the challenge in matching cognitive states across the species is a general problem that impinges on our understanding of human cognition informed by corresponding insights obtained from animal models.

It is obvious that for systematic study of attention-related modulations in monkeys, tasks that are easier and quicker to train animals to conduct are needed, including approaches that provide better control of or insight into behavioral strategies. For example, motivational reward incentive cues could be used to significantly speed up training and potentially provide better trial-by-trial experimental control in monkeys, as was shown by a study demonstrating visual stimulus category discrimination in tens of trials (Minamimoto et al. 2010). However, experimental conditions designed for monkeys might not always be optimal for assessing comparable attentional modulations in humans. The present study demonstrates that extrapolating information about neural mechanisms from animals to humans critically depends on cross-species studies matching cognitive status or, when this is not possible, seeking to understand the bases behind potential cross-species divergences in attentional influences.

The results of previous neurophysiological studies suggest that audio–visual selective attention tasks, and other active listening tasks, have quite diverse effects on neural responses in monkey auditory cortex (Osmanski and Wang 2015). During auditory tasks, auditory cortical neurons show both response enhancement and suppression effects, non-auditory responses and task-dependent effects (Hocherman et al. 1976; Brosch et al. 2015). The results of a few recent studies also suggest that the effects of active auditory tasks on responses in non-primary regions differ from those observed in primary auditory cortex (Atiani et al. 2014; Niwa et al. 2015). The present fMRI study showed that auditory attention effects are not homogenous across monkey STG. Further, the present fMRI results, together with those reported in a recent neurophysiological study (Lakatos et al. 2016), show that lapses in auditory selective attention strongly alter activation in macaque auditory cortex. Taken together, it is clear that a better understanding of the effects associated with attention-engaging tasks across different brain regions is needed. Although fMRI does not provide direct access to neuronal responses (Mukamel et al. 2005; Logothetis 2008), the present and another recent study in the visual modality (Patel et al. 2015) show that fMRI during attention-engaging tasks enables cross-species comparisons of large-scale brain activation. Both large-scale and neuronal-level measurements are important for establishing the common principles by which neural processes are modulated during active tasks.

### Do Task Performance Differences Explain Modulation of Auditory Cortex?

In the present study, task performance was better in the visual than auditory task in both monkeys and humans, as is also often the case in previous human and monkey auditory attention studies (Hocherman et al. 1976; Petkov et al. 2004; Rinne 2010). It could be argued, that in the present study, this difference in task performance affected the pattern of modulations observed in auditory cortical regions. However, a general behavioral difference would be expected to scale activations rather than cause nonuniform effects in auditory cortex, which is what we observed. Also, previous human fMRI studies show that auditory (spatial discrimination task, Rinne et al. 2012) or visual (Rinne 2010) task difficulty, as such, does not systematically modulate activations in auditory cortex. Moreover, the activation enhancements in anterior STG were largely overlapping when the A > V comparisons were conducted using HI (no significant difference in HI rates between auditory and visual task) or CR (CR rate was higher during visual than auditory task) trials. Thus, it is unlikely that task performance differences strongly contributed to the present activation enhancements in auditory cortex. A more parsimonious interpretation is that the enhanced activations in monkey and human auditory cortex are due to modulation of auditory processing during the active listening task, with lability in selective-attention performance altering attention-related effects inhomogenously across cortical regions.

## Conclusions

This study identifies key regions strongly modulated by audio–visual selective attention in the primate brain. To our knowledge, these monkey fMRI results show the first systematic modulation of cortical responses by an auditory attention-engaging task in nonhuman animals, with a certain level of correspondence in relation to the results in humans. Moreover, the present results inform and can be used to revise models of selective attention networks, and identify key regions where auditory and visual selective attention effects could be studied in more detail at the neuronal level. Comparisons between our results in humans and monkeys at first glance appeared to identify certain regional differences in how auditory attention modulates the activations in the monkey and human brain. However, additional analyses pointed to sources for “lost” auditory attentional modulations in the monkeys, revealing a comparable pattern of modulated brain regions as the one modulated by auditory attention in humans. Comparative studies in humans and animal models remain important for improving the accuracy of neurobiological models of human cognition.
